# COVID-19 Pandemic as the Beginning of a Golden Era for Telepsychiatry in Poland's Healthcare System

**DOI:** 10.3389/fpsyt.2020.555559

**Published:** 2021-01-13

**Authors:** Łukasz Zadka, Marcin Olajossy

**Affiliations:** ^1^Division of Histology and Embryology, Department of Human Morphology and Embryology, Wroclaw Medical University, Wroclaw, Poland; ^2^Polish Psychiatric Association, Warsaw, Poland; ^3^2nd Department of Psychiatry and Psychiatric Rehabilitation Medical University of Lublin, Medical University of Lublin, Lublin, Poland

**Keywords:** telepsychiatry, telehealth, e-health, COVID-19, Poland

## Background

The COVID-19 pandemic has had a tragic impact on the health and economy sectors of many countries in the world. The deepening social isolation resulting from limited interpersonal contact, the need for quarantine, overloaded healthcare professionals, and the increasing feeling of global fear, may lead to long-term deterioration in the mental health of societies and outweigh the losses of the current crisis (1).

The new coronavirus has also become an attractive topic for mass media outlets that are outdoing each other in informing the public about the current infection and death rates. At the beginning of March (2), a media campaign was launched in Poland against some patients with COVID-19. The published articles emphasized the fact that infected persons had returned from foreign travels to the country, which caused strong public outrage and accusations of deliberately bringing the virus to Poland. Stigmatization of infected people by mass media particularly affected unaware doctors returning to Poland after a vacation during that time period (3, 4). An avalanche of negative online comments caused by the published articles has led to threats against those physicians, and consequently even to suicide (5, 6). The hate wave is a well-known phenomenon that increases the risk of aggression and the percentage of committed suicides, that requires the use of appropriate strategies to prevent the spread of these adverse social impacts (7).

## Fear of the Unknown

Telepsychiatry is a term first introduced in 1973 by Dwyer (8), that in the current definition is a broad issue and refers to activities carried out with information and communications technology (ICT) for the provision or support of psychiatric services at a distance (9). Despite numerous reports of beneficial effects of e-platforms on mental health (9–11), in the Polish medical community it was a topic of little interest. One possible reason was the later acceptance of this therapy method, that emphasizes the fact that the Polish equivalent of the English definition “telepsychiatria” was first defined in 2003 and until that time remained a widely unknown issue (12). Wojtuszek et al. found, that two-thirds of patients had never heard of this term, and yet half of the respondents saw the usefulness of applying telepsychiatry. In relation to physicians, 84% of respondents have never dealt with the practice of telepsychiatry and 64% would not like its broader implementation in routine medical practice. The article points to administrative difficulties, the lack of relevant legal regulations, technological limitations, no payment for e-services, and security issues as major obstacles to the practice of telepsychiatry (13).

## The Snows of Yesteryear

One of the restrictions on the use of telemedicine in Poland was also the Medical Code of Ethics (*Kodeks Etyki Lekarskiej* – KEL). Its 9th Article indicates that a physician may only start the treatment thorough a physical examination of the patient. This article allows the possibility of distance treatment, but only in the case of an urgent need (14). The interpretation of that term remains controversial and may be interpreted differently by the physician giving the services, and otherwise by the medical court.

For Poland, the most common reasons for e-consultations are emergency incidents of mental health deterioration most often associated with anxiety disorders, followed by mood disorders (15). Moreover, e-interventions have also been shown to be beneficial to patients with schizophrenia and the therapeutic approach itself was positively evaluated by these patients (12). However, the 6th Article of KEL mentions the physician's freedom to choose the methods of follow-up that are considered most effective. It should be noted that e-services allow patients a significant reduction in travel costs (16). Here, we should refer to the 57th Article of the 2nd Act of KEL, which clearly states that the physician is responsible for choosing the form of diagnosis or therapy without putting the patient at an unreasonable expenditure (14). Therefore, there are no premises preventing the provision of telepsychiatry with regard to ethical concerns.

## The Metamorphoses

The position of the Polish government on the latest changes in the Law of December 5, 1996 on the profession of physicians and dentists should be considered as positive. The Act allows clinicians to carry out diagnostic and therapeutic activities through ICT (17). The law as a valid legal act dispels any doubts about the application of telemedicine in Poland. The positive impact on the position of these services in the Polish healthcare system has also the obligation to provide e-prescriptions using the recently introduced IT platform, which has been in place since January 8, 2020 (18). These changes contribute to the better understanding of the digitization of medical systems, that should lead to increased trust in the use of ICT services.

Before the most recent pandemic, Polish psychiatrists highlighted the lack of places for patients who required hospitalization in mental health units, which resulted in the inability to provide medical benefits in line with current needs. Child and adolescent psychiatry has been particularly hit by this problem, where the needs were greatest, and the level of underfinancing remained high. The pandemic crisis caused concern associated with the obvious determinative state of the health status of patients who were in remission and the expected acceleration of mental health problems in the general population. The Polish Psychiatric Association (PTP) and the national psychiatry consultant took the initiative by making appropriate statements addressed to both the Ministry of Health and the National Health Fund of Poland. National consultants are appointed by the Minister of Health from among specialists in particular fields and their duties include but are not limited to: the initiation of national epidemiological research, the forecast of health needs in their specific field, advising on the implementation of important tasks in the Polish healthcare system, and giving opinion and advice on tasks related to the training program of medical specialists. Adequate action by the PTP contributed to an immediate change in Polish legislation for the provision of on-line services. The latest PTP recommendations are summarized in Table 1.

**Table 1:** Characteristic of telepsychiatric services available in Poland.

**Subject**	**Description**
Service definition	A remote visit (e-visit, tele-visit, online visit)
Customer	Patient identification required (identity card), the need to provide patient's personal data (first and last name, address, national identification number (PESEL) or the guardian's data (for patients with intellectual disabilities or if incapacitated)
Service provider	Physician, psychologist, psychotherapist
Recommended form of service provision	Telephone conversation or video consultation that ensures encrypted transmission; video communicators are the preferred form (*empathic patient-physician relationship; possible observation of facial expressions, reactions and patient behavior*)
Requirements	Past medical history, current medical documentation, including the results of laboratory tests and/or neuroimaging findings the physician may ask the patient to send scans of documentation by an email or via SMS/MMS.
Responsibility	The consequences of providing incorrect, incomplete, false, misleading or otherwise incorrect data are the sole responsibility of the patient.
Restrictions	The examination cannot fully replace a direct medical or psychological examination; teleconsultation is allowed only if a direct examination cannot take place
Important reasons allowing the patient to be examined at a distance	Epidemiological threat, forced isolation of the patient or unavailability for other reasons
Conditions for refunding benefits	Provision of services using ICT systems at the place of providing benefits

*ICT, Information and communications technology; SMS, Short message service; MMS, Multimedia messaging service*.

*Based on recommendations of the Scientific Section of Telepsychiatry of Polish Psychiatric Association (19); Regulation of the Minister of Health of 16th March, 2020 (20)*.

## The Revolution - Telemedical Services Are Just as Important as an In-Person Visit

The Polish Chamber of Physicians and Dentists (*Naczelna Izba Lekarska*- NIL) at a meeting on July 24, 2020 adopted an act on the recognition of guidelines for the provision of telemedicine services along with the recommendation of its use by physicians and dentists as part of their profession. As highlighted, a significant contribution in the legislative work should be attributed to the statements made by Polish medical societies and recently implemented changes to the legal norms (21). Moreover, the Presidium of NIL called on the Minister of Health to undertake prompt steps leading to the introduction of proposed guidelines as an existing standard for the Polish healthcare system (22, 23). The appeal took into account the benefits of implementing telemedicine services into routine medical practice, as well as the negative impact of the COVID-19 pandemic which required adequate action, justifying the immediate use of ICT services.

Obviously, the provision of telemedicine services must be consistent with the Polish legal system. The emphasis was placed on the issue of the security of the patient's personal data and medical data, emphasizing the need to maintain medical confidentiality when performing e-services. Healthcare institutions providing e-services in the field of telemedicine are required to secure data transmission and enable optimal accessibility to such services for every recipient. This means ensuring appropriate system requirements, securing the network, and providing facilities with all electronic tools needed to enable e-consultation with a patient. With regard to medical practitioners, the appropriate amendments to professional liability related to the provision of telemedicine services have been taken into account, in light of which the use of telemedicine is not only highly recommended, but also mandatory when the patient's condition requires it. If a physician fails to provide e-services, when available and required, they could be held legally liable for malpractice. The healthcare entities employing medical staff are now responsible for their mistakes resulting from given e-services, if the employee was hired under an employment contract. In case of a contract physician, the liability rules will be enforced individually on the basis of the concluded contract.

A need to use telemedicine services during the COVID-19 pandemic was noticed, which was emphasized in the prepared guidelines as well as in the written communication to the Minister of Health. An additional interpretation was also introduced in relation to the previous rules of medical ethics, which previously limited the implementation of e-services, thus a physical examination of the patient is currently recommended only in cases where it is necessary to perform it. The following NIL directives, included in the guidelines, are particularly worth emphasizing:

- Telemedicine is a recognized method of patient care, and thus it can be treated as one of the standards of medical treatment.
- The medical practitioner should use the potential of telemedicine to realize the patient's rights.
- Telemedicine enables the implementation of individual patient rights in a new, digital way.
- Telemedicine advice should not be disregarded due to its remote form. The rules of professional, civil, and criminal liability for telemedicine services are the same as in the case of other services, and the recipient receives all rights pertaining to a patient.

The adopted guidelines constitute a decisive and important turnaround to the previously discussed limitations of the implementation of telemedicine services resulting from some articles of KEL.

## It is Too Soon for the Epilog

The updates incorporated into the Polish healthcare sector are moving in the right direction. In our opinion, previously hospitalized patients on long-term treatment will benefit the most. Many of them are often in contact by phone to provide expert support. There are still doubts to first-time treated patients, where the diagnosis, often in psychiatry, is based on medical history and may be difficult, thus actions taken will be at risk. However, it should be noted that in the face of the current pandemic, special care is required for patients with anxiety disorders. Panic and behavioral change due to the necessity of wearing gloves and medical masks to protect against the contamination by the new coronavirus may increase anxiety and polarize the current problems in the direction of a “coronaphobia” (24). The increased availability of e-services will also intensify the phenomenon of the deinstitutionalization of Polish psychiatry, will help patients gain access to medical specialists, and it will reduce the difficulties included with a limited number of hospital beds. Therefore, we look forward to the future with optimism.

## Author Contributions

ŁZ: study design, preparation of the manuscript in Polish and English, and selection of references, MO: correction of the Polish version of the manuscript and selection of references. All authors significantly contributed to the writing of the manuscript and approved its final version.

## Conflict of Interest

The authors declare that the research was conducted in the absence of any commercial or financial relationships that could be construed as a potential conflict of interest.
